# A Bayesian Framework for the Analysis and Optimal Mitigation of Cyber Threats to Cyber‐Physical Systems

**DOI:** 10.1111/risa.13900

**Published:** 2022-03-01

**Authors:** Piotr Żebrowski, Aitor Couce‐Vieira, Alessandro Mancuso

**Affiliations:** ^1^ International Institute for Applied Systems Analysis (IIASA) Laxenburg Austria; ^2^ Instituto de Ciencias Matemáticas Consejo Superior de Investigaciones Científicas Madrid Spain; ^3^ Department of Mathematics and Systems Analysis Aalto University Espoo Finland; ^4^ Department of Energy Engineering Politecnico di Milano Milan Italy

**Keywords:** Bayesian networks, cybersecurity, electric power grids, multiobjective optimization, risk management

## Abstract

Critical infrastructures are increasingly reliant on information and communications technology (ICT) for more efficient operations, which, at the same time, exposes them to cyber threats. As the frequency and severity of cyberattacks are increasing, so are the costs of critical infrastructure security. Efficient allocation of resources is thus a crucial issue for cybersecurity. A common practice in managing cyber threats is to conduct a qualitative analysis of individual attack scenarios through risk matrices, prioritizing the scenarios according to their perceived urgency and addressing them in order until all the resources available for cybersecurity are spent. Apart from methodological caveats, this approach may lead to suboptimal resource allocations, given that potential synergies between different attack scenarios and among available security measures are not taken into consideration. To overcome this shortcoming, we propose a quantitative framework that features: (1) a more holistic picture of the cybersecurity landscape, represented as a Bayesian network (BN) that encompasses multiple attack scenarios and thus allows for a better appreciation of vulnerabilities; and (2) a multiobjective optimization model built on top of the said BN that explicitly represents multiple dimensions of the potential impacts of successful cyberattacks. Our framework adopts a broader perspective than the standard cost–benefit analysis and allows the formulation of more nuanced security objectives. We also propose a computationally efficient algorithm that identifies the set of Pareto–optimal portfolios of security measures that simultaneously minimize various types of expected cyberattack impacts, while satisfying budgetary and other constraints. We illustrate our framework with a case study of electric power grids.

## 1 INTRODUCTION

Cyber‐physical systems, consisting of physical installations monitored and controlled by networks of electronic sensors and computers, are increasingly employed in a wide range of industries (Lee et al., 2015). A prominent example are smart electric power grids, which increase the efficiency and responsiveness of power systems, enabling a cheaper and more reliable power supply. However, developing critical infrastructures, such as power grids, into cyber‐physical systems exposes them to threats of a digital nature (Smith & Paté‐Cornell, 2018).

In recent years a dramatic increase has been seen in the quantity, diversity, and sophistication of cyberattacks, leading to significant economic losses (World Economic Forum, 2020). Cyberattacks can disrupt production processes: Notable examples include the 2010 Stuxnet attack on several Iranian uranium enrichment facilities (Nourian & Madnick, 2018) and the 2014 attack on the control systems of a German steel mill (Lee et al., 2014). Cyberattacks can cause financial losses, as was the case with the worldwide wave of ransomware attacks on thousands of companies in 2017 (Yaqoob et al., 2017). Attacks on critical infrastructure are particularly disruptive and costly, as shown by the 2015 attack on over 50 substations of the Ukrainian power grid, which caused the loss of 130 MW of load and a power outage of several hours for 225,000 users (Whitehead et al., 2017). Recently, a ransomware attack forced the Continental Pipeline to shut down its operations, resulting in substantial fuel supply disruptions across the southeastern United States (Sanger et al., 2021).

### 1.1 Review of Industry Practices and Literature

The increase in cyber threats to critical infrastructures prompted the development of industry guidelines, such as National Institute of Standards and Technology (2018), which aims to improve the security of these essential cyber‐physical systems. Cyber risks are typically assessed within the framework proposed by Kaplan and Garrick (1981), which characterizes risks in terms of triplets of undesired events, the likelihood of their occurrence, and their impacts. For prioritization and management of cyber threats, the bulk of the industry guidelines rely on risk matrices (see e.g., Electric Power Research Institute, 2015a). In this simple and intuitive method, threats are given pairs of ordinal ranks (e.g., low, medium, or high), based on expert judgment of the likelihood of their occurrence and of the severity of their impacts. The likelihood and impact rankings are then collapsed into a single priority ranking by assigning priority rankings to combinations of likelihood and impact rankings, for example, threats that have the highest ranking for likelihood and severity are considered as being top priority and the most urgent to focus on. The subjectivity of this procedure may, however, lead to incorrect risk prioritization (Cox, 2008; Duijm, 2015), and the sequential choices of mitigating actions based on it may result in a suboptimal portfolio of countermeasures (Allodi & Massacci, 2017). The recognition of deficiencies in standard practices has sparked active research on methods of cybersecurity risk assessment and management (Cherdantseva et al., 2016), ranging from qualitative to quantitative.

A better way to assess cyber risks is to use an analytical framework that reflects the nature of cyberattacks as multistage events. It is thus increasingly common to use attack trees in cybersecurity assessments ((see, e.g., Electric Power Research Institute, 2015b). An attack tree is a concise graphical representation of multiple possible ways of carrying out an attack, understood as sequences of an attacker's exploits leading to a breach in a cybersecurity system. Attack trees and related graphical models are widely used in security modeling (Kordy et al., 2014). They are convenient tools for analyzing system vulnerabilities (Byres et al., 2004; Ten et al., 2010) and in planning for deployment of countermeasures. For instance, Roy et al. (2010) discuss methods based on minimal cut sets in attack graphs and on minimization of expected loss; Serra et al. (2015) and Shelar and Amin (2017) use attack graphs to develop game‐theoretic approaches to finding optimal defence strategies; while Shameli‐Sendi et al. (2018) propose a method for dynamic deployment of the countermeasures that are least disruptive to the operations of the system.

As the complexity of cyberattacks increases, the quantitative frameworks of assessment and management of cyber risks shift from a score‐based description of likelihoods to a probabilistic one, as the latter allows for a meaningful combination of the likelihoods of atomic exploits into the likelihood of a successful multistage attack. Attack trees and attack graphs prove to be good foundations for probabilistic risk assessment models. Wang et al. (2008) developed a probabilistic metric for quantifying the likelihood of a multistep cyberattack, using attack graphs, whose nodes represent single‐step exploits, augmented with probabilities of single‐step exploits and the conditions required for those exploits to occur. Liu and Man (2005) replaced these probabilities with conditional probabilities of exploits represented by nodes, given the states of upstream nodes, thus turning an attack graph into a Bayesian network (BN). This allowed for the likelihood of the system compromise to be calculated as the attack unfolds by chaining conditional probabilities of single‐step exploits along the attack path. Peng Xie et al. (2010) discussed methods of turning attack graphs into BN, and argued that such networks can be used for real‐time monitoring of system vulnerabilities.

BNs are also a sound basis for quantitative methods of cyber risk management, particularly in the design of efficient portfolios of risk‐mitigating measures. Poolsappasit et al. (2012) use a Bayesian attack graph (BAG) as a model for the vulnerability to attacks of an ICT infrastructure and add to it a set of security countermeasures, which, when deployed, modify the conditional probabilities of successful attacks on nodes of the BAG. They propose a genetic algorithm to find a set of Pareto‐optimal sequences of countermeasure deployments that offer the best available balance between the cost‐effectiveness of the security measures and the reduction of expected losses in the event of an attack.

Objective selection is a key decision for developers of quantitative cyber risk management frameworks and a defining factor for optimal security policies. The scholarship with regard to finding optimal responses to cyberattacks focuses predominantly on tradeoffs between the expected losses borne by the system's operator in the event of cyberattack and the costs of cybersecurity responses—see, for example, Poolsappasit et al. (2012) and Serra et al. (2015). Although other objectives can also be considered, such as quality of service (Shameli‐Sendi et al., 2018), a cost–benefit analysis approach is dominant in the literature.

### 1.2 Contributions and Focus of the Article

The literature on cybersecurity is abundant, yet its main focus is on information and communicatinons technology (ICT) security. As far as cyber‐physical systems are concerned, especially critical infrastructures such as electric power grids, some aspects are not satisfactorily addressed. In this article, we address the following gaps:

1. The shortcomings of existing industry guidelines relying on expert judgment and the degree of arbitrariness in scoring systems used for assessing and managing cyber threats are well recognized. Yet, the development of intuitive and computationally efficient methods of quantitative risk analysis that combine expert judgment and statistical analysis of available data is still an open problem (Zio, 2009). BNs are considered to be a promising answer to this problem (Couce‐Vieira et al., 2017). Building an adequate graphical representation of the vulnerabilities of a cyber‐physical system that defines the structure of the BN may be difficult, however. Elicitation of the probabilities of atomic exploits is another difficulty that may limit the practical use of BNs in cybersecurity assessments. The possibilities of using the existing results of standard analyses, such as descriptions of cyberattack scenarios and scores of their likelihoods and impacts, as a basis for graphical probabilistic models are, in our opinion, both promising and underexplored.
2. We observe that in the literature, scenarios of cyberattacks tend to be analyzed separately, as if they were independent. In reality, however, an ongoing attack of one type may increase the chances of success of another kind of attack. In other words, attack trees representing different attack scenarios may share nodes. Such synergies between attack scenarios have nontrivial consequences for the overall assessment of the state of the system's security. Moreover, countermeasures deployed to mitigate one cyber threat may reinforce or interfere with the effects of measures countering some other threat. Therefore, a framework for cybersecurity assessment and management should account for such synergies and propose an optimal portfolio of countermeasures.
3. The majority of existing cyber risk management methods tacitly assume the perspective of the operator of a system and seek to minimize her potential financial losses in the event of a successful attack, while at the same time reducing the costs of maintaining the cybersecurity of the system. However, such a narrow perspective is insufficient in the context of cyber threats to critically important cyber‐physical systems because a successful attack may have multiple kinds of impacts on a variety of entities other than the system's operator. For instance, the impacts of a successful attack on a power grid may inflict financial losses on the utility company, jeopardize the safety of its workforce and installations, or even damage the economy or public and environmental safety (Electric Power Research Institute, 2015a). In our view, the impacts of such serious cyberattacks should not be subjected to cost–benefit analysis or otherwise aggregated, for example, measured as a sum of partial impact scores for different types of impacts, as proposed in Electric Power Research Institute (2015a). Instead, distinct impact dimensions should be treated explicitly. The management of cyber threats to critical infrastructure should be seen as a multiobjective optimization problem aiming at simultaneous minimization of adverse impacts in each of these dimensions.

To address these three gaps, we propose a framework for quantitative cyber risk assessment and management that features the following:

- a BN that can be composed of the attack trees of individual cyber‐threat scenarios, thus allowing a more holistic cybersecurity landscape to be mapped of a system that includes possible synergies among the threats it faces;
- an additional layer of leaf nodes that explicitly represent the distinct dimensions of impacts of cyber threats;
- an additional layer of root nodes that represent decisions about the deployment of individual cybersecurity measures;
- a computationally efficient explicit enumeration algorithm that finds the set of all Pareto‐optimal portfolios of security measures. The algorithm solves a multiobjective optimization problem, namely, the simultaneous minimization of expected impacts in all considered dimensions. This algorithm allows for budget and technical constraints (such as incompatibilities among different measures). It also allows for probabilistic constraints that limit occurrence probabilities for high‐impact tail events, which, in our opinion, satisfactorily addresses the well‐recognized controversy of focusing solely on expected values as the objectives in risk management (Kaplan & Garrick, 1981).

The framework proposed in this article is pertinent to any cyber‐physical system for which a Bayesian graphical representation of its vulnerabilities to cyberattacks can be built. Yet, asking for a graphical model to be available that reflects elements and operations of the system of interest, together with estimates of the probabilities of successful attack for all its nodes, is a tall order. Therefore, we begin with a discussion on how a BN representing the cybersecurity landscape of the system could be built with the help of existing security assessments that follow standard industry guidelines. Existing reports on the cybersecurity of particular cyber‐physical systems are confidential and systems operators are reluctant to share them with the research community. One of the few exceptions to this general trend are the publicly available assessments of cybersecurity of the U.S. electric power grids, published by the Electric Power Research Institute (EPRI) in the form of National Electric Sector Cybersecurity Organisation Resource (NESCOR) reports. The availability of these reports and the critical importance of electric power grids make analysis of the security of these large‐scale cyber‐physical systems a convenient and highly relevant illustrative case example, to which we will be referring throughout the article.

### 1.3 Structure of the Article

In Section 2, we briefly introduce the reader to the NESCOR framework for assessing cyber threats to electric power grids, which is based on attack scenario analysis employing detailed attack trees. We observe that individual attack scenarios may not be independent. To illustrate this, we demonstrate that attack trees representing different scenarios of attacks on the advanced metering infrastructure (AMI) of a power grid share nodes, which indicates synergies among these scenarios. To capitalize on this observation, we demonstrate how these attack trees can be merged into a larger integrated attack graph that represents the overall exposure of the system to cyberattacks better than a set of individual attack trees. In Section 3, we discuss how the integrated attack graph can be turned into a BN, which is the cornerstone of the quantitative cyber risk analysis and management framework proposed in this article. We begin with the mathematical definition of the BN and a short discussion of its properties. Next, we address the practical question of eliciting conditional probability tables (CPTs) for the BN‐based model that represents cyber threats to the system of interest. In Section 4, we formulate the multiobjective optimization problem of finding Pareto‐optimal portfolios of mitigation measures and discuss the implicit enumeration algorithm used to solve it. We demonstrate this method using the case problem of improving the cybersecurity of AMI. In addition, we propose the core index as a useful tool for deciding on which of the Pareto‐optimal portfolio of measures to implement. Finally, in Section 5 we discuss the strengths, weaknesses, and applications of the proposed method, as well as its possible extensions, and present our conclusions in Section 6.

## 2 BUILDING A GRAPHICAL REPRESENTATION OF CYBERSECURITY LANDSCAPES FROM INDIVIDUAL ATTACK TREES

Attack trees and similar concepts are popular tools in the cybersecurity practice and literature. In this section, we demonstrate how individual attack trees can be merged into a larger attack graph that gives a broader perspective of various, potentially synergistic cyber threats. As a case example we use attack trees representing scenarios of cyberattacks on the AMI of a power grid, which were analyzed in NESCOR reports on the cybersecurity of electric power grids.

### 2.1 Overview of the NESCOR Practice

The NESCOR guidelines developed by Electric Power Research Institute (2015a) describe over 120 cybersecurity failure scenarios, understood as potential but realistic events in which the failure to maintain confidentiality, integrity, and/or availability of the system cyber assets creates a negative impact on the generation, transmission, and/or delivery of power.

The NESCOR analysis (Electric Power Research Institute, 2015a) recognizes that the impacts of cybersecurity failures on electric power grids are of a multifaceted nature and may afflict a variety of entities. Thus, 15 impact criteria are proposed, which can be understood as distinct risk dimensions. table 3 lists these criteria, together with a severity scoring system for each of them. The overall impact score, however, is calculated as a sum of these partial impact scores, which reflects neither the multidimensionality of impacts, nor the potential tradeoffs between them.

**FIGURE 1:**
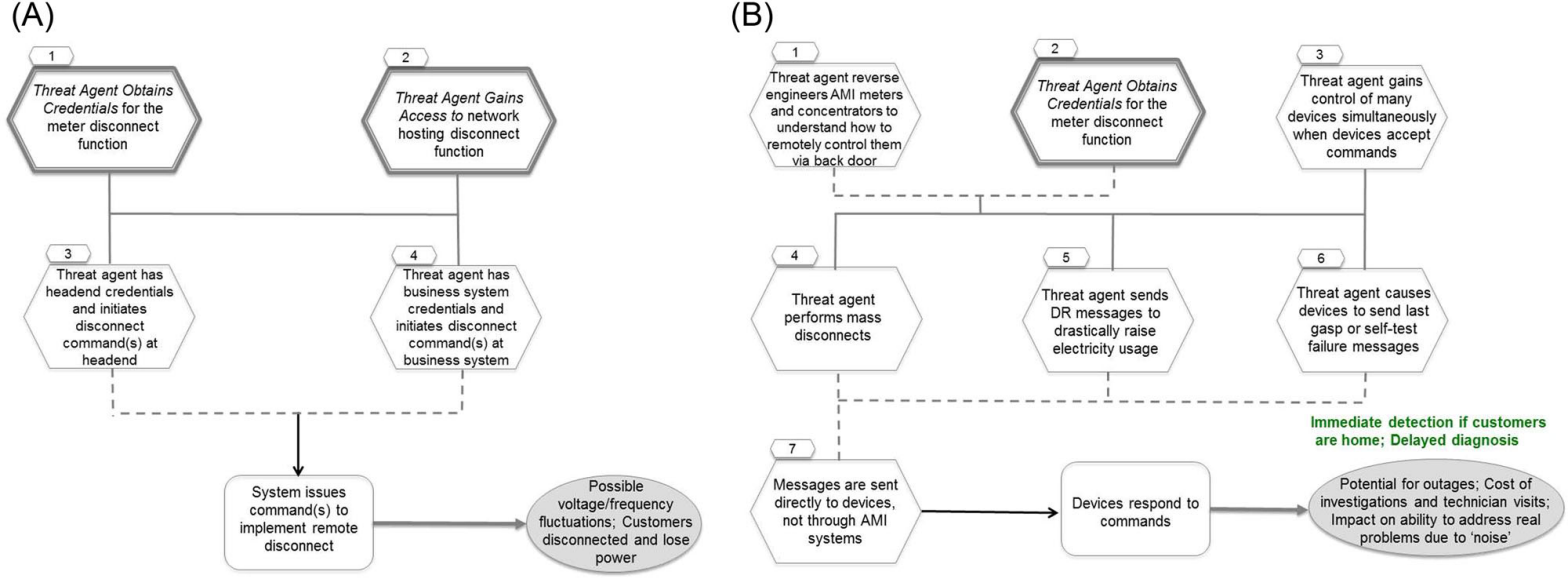
Attack graphs for (a) invalid disconnect messages to meters impact customers and utility (AMI.9) and (b) reverse engineering of AMI equipment allows unauthorized mass control (AMI.27) *Source*: Electric Power Research Institute (2015b).

The subsequent NESCOR report (Electric Power Research Institute, 2015b) provides more detailed descriptions of selected cybersecurity failure scenarios, as well as their graphical representations in the form of attack trees, examples of which (further discussed in the next subsection) are displayed in Figs. 1(a) and (b). Each attack tree represents chains of conditions (marked as hexagonal nodes) or their sequences (common subtrees, marked as hexagons with thick borders), the logical combinations of which (solid and dashed lines representing AND and OR operators, respectively) lead to a failure (rectangular node) and subsequent system responses and impacts (oval node).

A graphical representation of a cybersecurity failure scenario provides insights into its mechanisms and allows for better appraisal of the likelihood of its occurrence, which NESCOR guidelines (Electric Power Research Institute, 2015a) propose to assess according to five criteria related to the difficulty of creating and exploiting conditions leading to a failure. The corresponding partial likelihood scores are calculated according to the scoring systems presented in table 4 and then the overall likelihood score is calculated as a sum of the partial scores. Such an overall likelihood does not follow the arithmetic of probabilities, however. As a consequence, likelihoods of distinct events cannot be meaningfully combined and thus the dependencies or correlations between different failure scenarios cannot be quantified.

Nevertheless, we observe that some of the NESCOR attack trees share nodes or common subtrees, which indicates synergies between the failure scenarios they represent. For instance, the attack trees presented in Figs. 1(a) and (b) share the common subtree Threat agent obtains credentials for the meter disconnect function, which means that if this condition is satisfied for one of the two scenarios, the other failure scenario is automatically more likely to materialize.

As already explained, the NESCOR framework for the assessment and management of cyber risks does not allow full advantage to be taken of this observation. Elements of this framework, however, can be used to build a more holistic picture of potentially interlinked cyber risks. First, by taking a union of attack trees (i.e., merging them according to the shared nodes), we can better appraise the causal relationships between the failure scenarios represented by these trees. Second, adding a layer of leaf nodes representing different impact dimensions allows us to gain a better understanding of the consequences of synergistic failure scenarios, as the realization of one scenario not only has immediate impacts but also increases the likelihood of other failures with potential impacts down the line. Finally, for each attack tree presented in Electric Power Research Institute (2015b), a list of mitigation measures that reduce the likelihood of the occurrence of specific conditions (nodes of the tree) is usefully supplied. We can thus add a set of nodes representing mitigation measures to our expanded attack graph. This gives us a better appreciation of how individual measures help in mitigating synergistic failures and allows us to select efficient portfolios of mitigation measures.

### 2.2 Example Attack Graph for Security of AMI Infrastructure

For an illustrative case example, we use information from NESCOR studies (Electric Power Research Institute, 2015a, 2015b) to build an attack graph representing cyber threats to the AMI of electric power grids. The AMI includes a large number of smart power meters, which allow real‐time monitoring of customers' power consumption. The AMI also helps to manage the power system, for example, through demand–response actions. However, the wide dispersion and lack of physical protection of AMI devices raise many security concerns, as they allow for two‐way communication with traditionally self‐contained and centralized power supply systems and thus open up the possibility of disruption to their operations.

**FIGURE 2:**
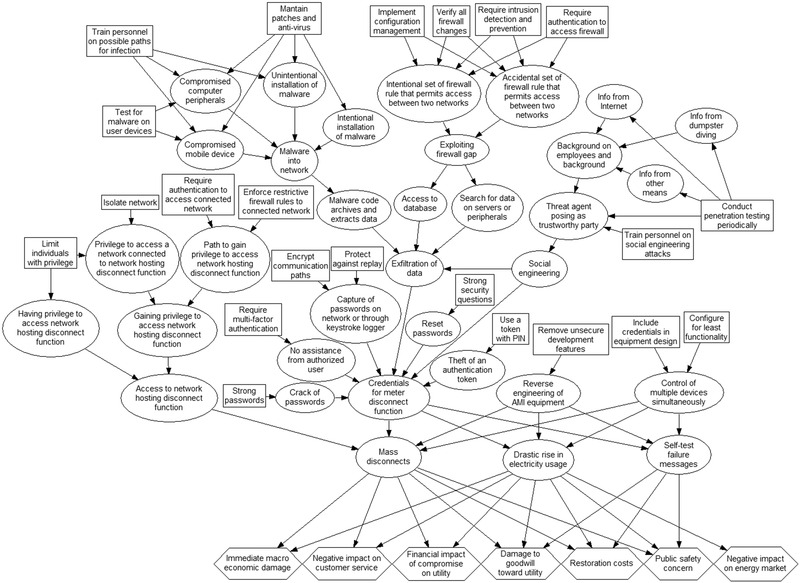
Attack graph (influence diagram) encompassing attack trees (and their common subtrees) for scenarios of cyberattack on the advanced metering infrastructure (AMI) of an electric power system: invalid disconnect messages to meters impact customers and utility (AMI.9) and reverse engineering of AMI equipment allows unauthorized mass control (AMI.27). The oval nodes represent individual exploits of the attacker, while rectangular nodes indicate mitigation measures that can reduce the success probabilities of exploits. The relevant impact dimensions are represented by hexagonal nodes

Electric Power Research Institute (2015b) provides a detailed description of six AMI‐related cybersecurity scenarios. To simplify our example, we focus on two of them: invalid disconnect messages to meters impact customers and utility (AMI.9) and reverse engineering of AMI equipment allows unauthorised mass control (AMI.27). The attack trees for these scenarios are depicted in Figs. 1(a) and (b), respectively. To build an integrated attack graph for these two scenarios, we merge their attack trees and the six common subtrees they contain: threat agent obtains credentials for system or function; threat agent uses social engineering; threat agent gains access to network; threat agent exfiltrates data; authorized employee brings malware into system or network; and threat agent exploits firewall. The resulting attack graph (represented as an influence diagram) is displayed in Fig. 2. To distinguish it visually from traditional attack trees, we have changed the shapes of its nodes. Oval nodes represent the events of the scenarios being considered, 1 while directed arcs indicate causal dependency between them. A layer of seven diamond‐shaped nodes at the bottom of the graph represents the relevant impact dimensions (criteria) for the scenarios in question. The rectangular nodes represent 22 mitigation actions, identified in Electric Power Research Institute (2015b) and listed in tables 5– 10, that could be taken to reduce risks related to the scenarios being studied. The arrows pointing from each of these decision nodes indicate which events in the considered scenarios are rendered less likely by the deployment of the corresponding mitigation measures. Fig. 2 shows synergies between the two failure scenarios under consideration as shared uncertainty nodes (e.g., *Credentials for meter disconnect function*) and indicates that mitigation measures can be deployed strategically to take advantage of these synergies.

## 3 A BN MODEL FOR ANALYSIS AND MANAGEMENT OF CYBER RISKS

In the previous section we demonstrated how attack trees, commonly used in the standard framework of cyber risk analysis, can be combined into an attack graph, like the one in Fig. 2, to help uncover potential synergies between attack scenarios. The standard framework does not offer ways of quantitatively describing these synergies, however, since the likelihood scores of considered scenarios cannot be meaningfully combined (as explained in Section 2.1). Therefore, to allow for quantitative risk analysis that makes use of the attack graph we need to turn it into a graphical probabilistic model of BN.

### 3.1 Definition of the BN

A BN consists of (1) a set of nodes arranged into a directed acyclic graph (DAG), whose edges represent causal links between the nodes; and (2) a probability distribution defined over this set of nodes. BNs considered in this work have the following three types of nodes:

BNs considered in this work have the following three types of nodes:

- *Uncertainty nodes* (drawn as circles), which represent stage events of attack scenarios and correspond to respective condition nodes in attack trees. We label them by integers from 1 to the number of uncertainty nodes N. To each i∈{1,…,N} a discrete random variable $X_{i}$ is assigned, which takes values from a finite set $S_{i}$ of possible states of the node i, including one representing no occurrence. The distribution of $X_{i}$ is dependent on the values of its parent nodes $pa(X_{i})$, that is, nodes with an edge pointing to $X_{i}$ and represented by a CPT.
- *Decision nodes* (drawn as rectangles), which represent decisions on the deployment of available mitigation measures $a_{1},\text{…},a_{M}$. For each j∈{1,…,M} the value $z_{j}$ of j‐th decision node is either 1 for deployment of $a_{j}$ or 0 for no deployment. $z_{j}$ is always known and is a parameter of the probability distribution over each of the child nodes of $a_{j}$, that is, nodes being pointed to by an edge starting from $a_{j}$. The binary vector $z=(z_{1},\text{…},z_{M})$ represents the states of all decision nodes in the network and is conveniently interpreted as a portfolio of deployed mitigation measures.
- *Value nodes* (drawn as hexagons), which represent the impacts of failure scenarios in the K dimensions considered (corresponding to impact criteria). For each k∈{1,…,K}, the state of the k‐th value node $V_{k}=v_{k}\left(pa\left(V_{k}\right)\right)$, where $v_{k}$is a real‐valued deterministic function of states of the parent nodes $pa(V_{k})$ of $V_{k}$ and represents the k‐th impact criterion (with value 0 for *no impact*).

**FIGURE 3:**
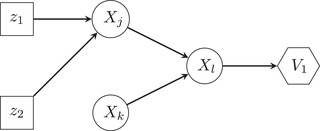
Example of a Bayesian network with decision nodes with states $z_{1}$ and $z_{2}$ representing states of deployment of measures $a_{1}$ and $a_{2}$, uncertainty nodes j, k, l whose states are represented by discrete random variables $X_{j},X_{k},X_{l}$ and value node $V_{1}$

In this article, we are concerned with designing a portfolio of static mitigation measures that reduce the likelihood of success of potential cyberattacks (not in dynamic response to ongoing attacks). BNs appropriate for this type of problem have the following structure: (1) All decision nodes are root nodes (i.e., ones that have no parents) and thus do not depend on any uncertainty nodes; (2) Uncertainty nodes are arranged in several layers, some being root nodes, some having multiple parents; (3) All leaf nodes (i.e., ones having no child nodes) are value nodes, and no value node can have a child node. An example network of this structure is displayed in Fig. 3. BNs of this type are also called influence diagrams (Jensen, 2001).

As the states of decision nodes are known *a priori* and the states of value nodes are deterministic functions of the states of their parent uncertainty nodes, the probability distribution over the whole DAG is determined by the probability distribution over the set of its uncertainty nodes. More precisely, given the portfolio of measures *z*, the joint probability distribution of $\left(X_{1},\text{…},X_{N}\right)$ is composed of their corresponding CPTs according to the chain rule:

$$P\left(X_{1},\text{…},X_{N}|z\right)=\prod_{i=1}^{N}P\left(X_{i}|X_{i},z\right),$$

where $X_{i}=pa\left(X_{i}\right)\cap \left(X_{1},\text{…},X_{N}\right)$ is the set of all uncertainty nodes that are parents of $X_{i}$ and $P(X_{i}|X_{i},z)$ is given by the CPT of $X_{i}$.

Let $\Delta_{i}$ be the set of all possible states of $X_{i}$, that is, $\Delta_{i}={⨂}_{j:X_{j}\in X_{i}}S_{j}$, where ⨂ denotes the Cartesian product. Then, for any state $s\in S_{i}$

(1) $$P\left(X_{i}=s|z\right)=\sum_{\delta \in \Delta_{i}}P\left(X_{i}=s|X_{i}=\delta ,z\right)P\left(X_{i}=\delta |z\right).$$

By the d‐separation property of BNs (Jensen, 2001), the joint probability $P(X_{i}=\delta |z)$ can be factorized as

(2) $$P\left(X_{i}=\delta |z\right)=\prod_{j=1}^{\left|\delta \right|}P\left(X_{i}^{\left(j\right)}=\delta^{\left(j\right)}|z\right),$$

where |δ| is the number of elements of δ, while $X_{i}^{\left(j\right)}$ and $\delta^{\left(j\right)}$ stand for j‐th elements of $X_{i}$ and δ, respectively. Thus, with use of (1) and (2) we can calculate $P(X_{i}=s|z)$ from CPTs of the nodes upstream of $X_{i}$.

Similarly, we can calculate the probability distribution at impact node $V_{k}$. Let $X_{k}=pa\left(V_{k}\right)$ be the vector of parent uncertainty nodes for $V_{k}$ and let $\Delta_{K}={⨂}_{j:X_{j}\in X_{K}}S_{j}$ be the set of all possible states of $X_{k}$. Recall that $V_{k}=v_{k}\left(X_{k}\right)$, where $v_{k}$ is a deterministic function. Then for any $\delta \in \Delta_{k}$

$$P\left(V_{k}=v|X_{k}=\delta ,z\right)=1\left(v_{k}\left(\delta \right)=v\right),$$

where 1(A) is an indicator function taking value 1 if the expression A is true and 0 otherwise. Plugging this into (1) we get the following distribution:

(3) $$P\left(V_{k}=v|z\right)=\sum_{\delta \in \Delta_{K}}1\left(v_{k}\left(\delta \right)=v\right)P\left(X_{k}=\delta |z\right).$$

### 3.2 Developing Attack Graphs Into BNs

A BN may be a potent tool for monitoring and managing the cyber risks of a system but building one that appropriately represents the security challenges of the system often proves not be an easy task. First, a DAG representing system vulnerabilities must be specified. Methods of automated generation of attack trees, like the one developed by Johnson et al. (2017) for ICT systems, may make this process more manageable, but it usually requires substantial amounts of work and expert knowledge. In the context of the cybersecurity of electric grids, graphical representation of system vulnerabilities like the exemplary DAG in Fig. 2 can readily be built from the NESCOR attack trees (Electric Power Research Institute, 2015b), as discussed in Section 2.2. Generally, building an appropriate DAG underlaying the BN model, although tedious, is technically feasible, and in this section we assume that such DAG is given.

To turn a DAG into a BN, one needs to specify CPTs for its uncertainty nodes and impact functions for its value nodes. Probability distributions for nodes representing initial exploits opening attacks (i.e., uncertainty nodes $X_{i}$ with $X_{i}=\emptyset$) can be estimated based on data (e.g., system logs), or, in the absence of reliable data, based on more subjective expert knowledge. The effects of the deployment of mitigation measures on the probability distributions of the uncertainty nodes that represent the vulnerabilities addressed by these measures can be estimated in a similar way. Specifying CPTs for downstream uncertainty nodes is more challenging, as it involves determining the values of multiple parameters of distribution for all combinations of states of parent nodes. The number of these parameters may run into the thousands for large networks, making it unfeasible to elicit their values from experts, while lack of reliable data often hinders their estimation. What could be reliably specified by the experts, however, are the logical relationships between the conditions that open the ways for further exploits. Such logical structures, like the combinations of AND and OR operators represented by the NESCOR attack trees, make the derivation of CPTs a more straightforward task.

Continuing the example from Section 2.2, Table 1 displays a CPT representing logical combinations of upstream conditions leading to the event *Threat agent performs mass disconnects* (Fig. 1(b)). Other examples of this approach can be found in the literature. For instance, in a similar fashion, Bobbio et al. (2001) represent combinations of AND and OR operators as CPTs in order to develop fault trees into BNs. They also introduce noisy‐ANDs and noisy‐ORs (and their CPT representations), which are randomized versions of their classical counterparts. Khakzad et al. (2013) adapt this approach to the computation of CPTs for mapping bow‐tie failure models into BNs, while Peng Xie et al. (2010) apply it in the context of modeling cybersecurity. Noisy‐ANDs and noisy‐ORs are particularly useful for modeling the escalation of cyberattacks whose consecutive stages are not automatically achieved or may fail even if the necessary conditions for them have occurred.

**TABLE 1:** Conditional Probability Table Based on Binary States

			Threat Agent Performs	
Threat Agent Reverse	Threat Agent	Threat Agent Gains	Mass Disconnects	
Engineers AMI Equipment	Obtains Credentials	Control of Devices	Occurrence	No Occurrence
Occurrence	Occurrence	Occurrence	1	0
		No occurrence	0	1
	No occurrence	Occurrence	1	0
		No occurrence	0	1
No occurrence	Occurrence	Occurrence	1	0
		No occurrence	0	1
	No occurrence	Occurrence	0	1
		No occurrence	0	1

As an alternative, Frigault et al. (2008), Peng Xie et al. (2010), Poolsappasit et al. (2012), and Zhang et al. (2015) propose methods of deriving the success probabilities of exploits from the Common Vulnerability Scoring System (CVSS), used widely in security assessments of cyber‐physical systems. Exploit probabilities derived in this way can be employed in our framework. Estimates of exploit probabilities based on CVSS may, however, be unreliable due to the arbitrariness and ambiguity of the scoring system (Allodi & Massacci, 2014; Spring et al., 2018). The use of CVSS assessments should therefore be considered only if there is no other practical basis for estimating the success probabilities of exploits.

In the literature on BN models of system security and reliability, the focus is mainly on the networks with binary uncertainty nodes, that is, ones that only have states of *occurrence* or *no occurrence*. For a more realistic representation of the possible courses of an attack, it may be advantageous to allow for uncertainty nodes having more than two states. For instance, conditions *Threat agent reverse engineers AMI equipment* and *Threat agent obtains credentials* considered in Table 1 are binary in nature, but *Threat agent gains control of devices* may have multiple states representing the scale of the attacker's exploit. The exemplary CPT in Table 2 is a modification of the CPT in Table 1 where we allow the threat agent to gain control of no devices, a few, a moderate number, or a high number of devices, resulting in different scales of disconnects, ranging from zero to over 100 MW loss of load. Importantly, the values in Table 2 are not meant to be representative of any particular electric system and were chosen for demonstration purposes. Combinations of nonbinary conditions can also be represented as CPTs, for instance, using the noisy‐MAX operator proposed in Bobbio et al. (2001).

**TABLE 2:** Conditional Probability Table Based on Multiple States

Threat Agent	Threat Agent	Threat Agent				
Reverse Engineers	Obtains	Gains Control	Threat Agent Performs Mass Disconnects [MW]			
AMI Equipment	Credentials	of Devices	*No Occurrence*	(050]	(50100]	>100
Occurrence	Occurrence	None	1	0	0	0
		Few	0.6	0.4	0	0
		Moderate	0.4	0.2	0.4	0
		High	0.3	0.1	0.2	0.4
	No occurrence	None	1	0	0	0
		Few	0.6	0.4	0	0
		Moderate	0.4	0.2	0.4	0
		High	0.3	0.1	0.2	0.4
No occurrence	Occurrence	None	1	0	0	0
		Few	0.6	0.4	0	0
		Moderate	0.4	0.2	0.4	0
		High	0.3	0.1	0.2	0.4
	No occurrence	None	1	0	0	0
		Few	1	0	0	0
		Moderate	1	0	0	0
		High	1	0	0	0

**FIGURE 4:**
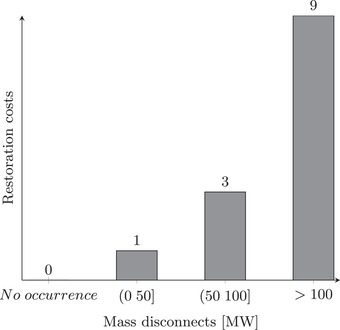
Illustrative impact scores for “Restoration costs”

The final component of a BN model is the set of functions $v_{k}$, k=1,…K representing the severity of impacts of a cybersecurity failure in each of the K impact dimensions. Existing impact‐scoring systems, like the one developed by NESCOR (see Table S1), are a convenient basis for specifying these functions. For instance, continuing with our example from Section 2.2, the uncertainty node representing the event *Threat agent performs mass disconnects* may have the states *no occurrence*, (0,50] MW, (50,100] MW, or >100 MW, which can be mapped to the *Restoration costs* score of 0, 1, 3, or 9, respectively (cf. Fig. 4).

As discussed above, a broad range of methods of developing attack graphs into BNs is available in the literature, which makes it feasible to build a BN representation of the cybersecurity landscape for the given cyber‐physical system. This, however, requires considerable effort and an intimate knowledge of the system of interest, which is usually available only to its operators. We will return to the practical aspects of real‐world applications of the proposed framework in the “Discussion” section. For now, we assume that a BN model of the system's vulnerabilities is available, and, in the next section, we turn our attention to the problem of strategically deploying a portfolio of measures that efficiently mitigate cyber threats to the system of interest.

## 4 THE MULTIOBJECTIVE OPTIMIZATION MODEL FOR SELECTING PORTFOLIOS OF SECURITY MEASURES

Our aim is to select efficient portfolios of security measures that, when deployed, minimize the expected impacts of cybersecurity failures and, at the same time, keep the probability of catastrophic impacts within limits deemed acceptable. For a portfolio z, the expected impact in the k‐th impact category can be computed with the use of formulas (2) and (3) as

$$E\left(V_{k}\right)\left(z\right)=\sum_{\delta \in \Delta_{k}}v_{k}\left(\delta \right)P\left(X_{k}=\delta |z\right)=\sum_{\delta \in \Delta_{k}}v_{k}\left(\delta \right)\prod_{j=1}^{\left|\delta \right|}P\left(X_{k}^{\left(j\right)}=\delta^{\left(j\right)}|z\right),$$

where $P(X_{k}^{\left(j\right)}=\delta^{\left(j\right)}|z)$ can be computed from CPTs using formulas (1) and (2).

### 4.1 Including Probabilistic, Technical, and Budget Constraints

**FIGURE 5:**
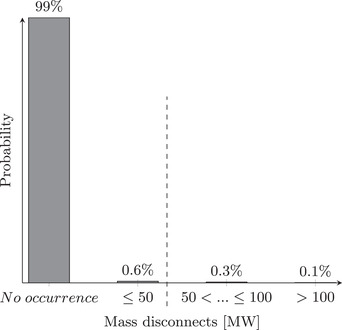
Illustrative probability distribution for mass disconnects

Focusing on the minimization of expected impacts in risk management has some well‐recognized pitfalls (Kaplan & Garrick, 1981), as it may lead to the selection of measures that reduce the more likely but less severe impacts, while not protecting against rare but potentially catastrophic ones. To counter this unwanted effect, probabilistic constraints may be imposed by stipulating that the deployment of measures must keep the occurrence likelihood of certain critical events below a selected threshold. Continuing our AMI example from Section 2.2, we may demand, for instance, that disconnects of 50 MW or greater can occur with a probability of at most 0.5% (see Fig. 5).

More formally, for each uncertainty node $X_{i}$ we may specify a set of critical states ${\tilde{S}}_{i}\subset S_{i}$ and a threshold probability $\alpha_{i}$ and demand that portfolio z satisfies the probabilistic constraint

(4) $$\sum_{s\in {\tilde{S}}_{i}}P\left(X_{i}=s|z\right)\leq \alpha_{i}.$$

Importantly, probabilistic constraints can also cap the severity of impacts at levels desired by system operators, with a probability deemed by them to be sufficiently high. Indeed, as the states of each value node representing certain impact dimensions are determined by the states of its parent uncertainty nodes, it is straightforward to select probabilistic constraints for parent nodes that, jointly, keep the probability of catastrophic impacts below the desired level.

There may also be technical constraints limiting the set of feasible portfolios of mitigation measures. For example, measures $a_{i}$ and $a_{j}$ may be incompatible and could not be deployed together. Portfolios not containing both of these measures at the same time must satisfy the inequality

$$z_{i}+z_{j}\leq 1.$$

Similarly, measures $a_{i}$ and $a_{j}$ may be effective only if deployed together. Such a constraint is formally represented as

$$z_{i}-z_{j}=0.$$

Last but not least, in most applications, the budget B available for maintenance of the system's security is limited, and the cost of feasible portfolios of measures must not exceed it. The budget constraint may be expressed as

$$\sum_{j=1}^{M}z_{j}c_{j}\leq B,$$

where $c_{1},\text{…},c_{M}$ are the costs of deployment of the available mitigation measures $a_{1},\text{…},a_{M}$.

Notice that technical and budget constraints are linear in z, which makes them very tractable. On the other hand, probabilistic constraints (4) are, in general, nonlinear, which may add significant difficulty to the multiobjective optimization problem. Observe, however, that if at most one measure from the set $A_{i}$ of all measures applicable to the node $X_{i}$ is deployed, then we can write

$$P\left(X_{i}=s|z\right)=\sum_{j|a_{j}\in A_{i}}z_{j}P\left(X_{i}=s|z\right)$$

as $z_{j}=1$ for at most one j such that $a_{j}\in A_{i}$ and all the rest is zero. Hence, the probabilistic constraint (4) becomes linear in z if $A_{i}$ contains only mutually exclusive measures—which we will assume without any loss of generality, as a combination of measures can be regarded as a new measure.

### 4.2 Optimization Algorithm for Identifying Pareto Nondominated Portfolios

The constraints shaping the set of feasible portfolios $Z_{F}$ usually do not allow for maximal possible reductions of all types of impacts at the same time, and compromises have to be made. It is therefore rational to focus only on the most efficient attainable tradeoffs, represented by the Pareto nondominated portfolios. Formally, a portfolio z dominates a portfolio $z^{\prime }$, denoted as $z⪰z^{\prime }$, if and only if $E\left(V_{k}\right)\left(z\right)\leq E\left(V_{k}\right)\left(z^{\prime }\right)$ for all criteria k=1,…,K and $E\left(V_{k_{0}}\right)\left(z\right)<E\left(V_{k_{0}}\right)\left(z^{\prime }\right)$ for at least one criterion $k_{0}\in 1,\text{…},K$. Portfolio z is then a rational choice over $z^{\prime }$ since it is better in reducing at least one type of impact while performing at least as well as $z^{\prime }$, according to all the other impact criteria. A portfolio $z^{*}\in Z_{F}$ is called Pareto nondominated, or Pareto‐optimal, if there is no other $z\in Z_{F}$ such that $z⪰z^{*}$. The set $Z_{ND}$ of all Pareto‐optimal portfolios is called a Pareto front.

To summarize, our problem of designing portfolios of measures that minimize the expected risks from cyberattacks can be formulated as the following multiobjective optimization problem. We aim to find portfolios belonging to the Pareto front $Z_{ND}$ given: (1) the BN representing the cybersecurity vulnerabilities of the system and options of measures to mitigate them; (2) the potential impacts of cyberattacks; (3) the costs of available security measures; and (4) the budget, technical, and probabilistic constraints shaping the set $Z_{F}$ of feasible portfolios of measures.

The Pareto front for this problem can be computed by adapting the explicit enumeration algorithm, developed by (Liesiö et al., 2008) and extended by Mancuso et al. (2017) to design portfolios of safety measures for nuclear power plants. Given a list of *M* possible mitigation measures, the algorithm executes an efficient search over 2*M* possible portfolios. It starts with an empty portfolio and adds consecutive measures from the list, updating the list of portfolios that are nondominated by any of the ones already explored. If the current portfolio becomes unfeasible after a new measure is added, the whole branch of portfolios containing the current one is excluded from the search space, and the measure is removed. Then the search continues as attempts are made to add the next measure from the list.^2^

During the search, the explicit enumeration algorithm excludes large parts of the search space that will certainly not contain feasible solutions. This makes it computationally efficient. A standard laptop can perform searches over a space of portfolios containing up to 40 measures, which is a realistic problem size for many applications. For larger problems, a viable alternative to computing the whole Pareto front with explicit enumeration is to approximate it using genetic algorithms at a lower computational time (Coello et al., 2007).

### 4.3 Core Index as a Guide for Selection of Pareto‐Optimal Portfolios of Mitigation Measures

The Pareto front $Z_{ND}$ usually consists of a large number of nondominated portfolios, which makes it difficult to decide which one to select. A decision support tool, like the one in Couce‐Vieira et al. (2017), can aid in the selection of the most desirable portfolio. Yet, it still requires preferences to be elicited from system operators. This task can be made easier by reducing the number of options to be considered. A useful guide for such a reduction is the core index CI(a) of a measure a (Liesiö et al., 2008), defined as

$$CI\left(a\right)=\frac{\left|\{z^{*}\in Z_{ND}|z_{a}^{*}=1\}\right|}{|Z_{ND}|}.$$

A value of CI(a) close to 1 means that the measure *a* is included in the majority of nondominated portfolios and can be regarded as belonging to the core of measures shared by the bulk of portfolios on the Pareto front. One may thus focus on nondominated portfolios containing measures with high CI(a) values and, by so doing, reduce the problem of selecting the most desirable portfolio to a deliberation over the inclusion of measures with lower CI. Moreover, when constraints shaping the Pareto front $Z_{ND}$, like the available budget, change, portfolios may be preferred that include the measures for which CI is stable.

### 4.4 Example of Optimizing a Portfolio of Measures That Reduces Cyber Risks to the AMI

We conclude this section with a demonstration of the potential of the Bayesian framework introduced above by applying it to the problem of improving the cybersecurity AMI system, used as an illustration throughout this article (see Section 2.2). The graphical representation of this problem is the DAG displayed in Fig. 2. The probability distributions over the uncertainty nodes of this DAG have been set accordingly in line with the information provided in NESCOR documents (Electric Power Research Institute, 2015a, 2015b) and using the NESCOR likelihood scoring system (see Table S2) as a guide. NESCOR documents, however, do not provide sufficient information to fully specify CPTs for the uncertainty nodes. We have therefore assumed illustrative values that yield a consistent probability distribution over the DAG but are not representative of any existing AMI system. Similarly, we use NESCOR impact scores shown in Table S1 to define the impact functions determining the states of value nodes (see the illustrative example of Restoration costs scores at the end of Section 3.2). Finally, decision nodes represent the list of 22 mitigation measures proposed in NESCOR studies, which are shown in Tables S3–S8, together with assumed illustrative costs for their deployment.

The search space for this problem contains $2^{22}$ possible combinations of measures, but the set of feasible portfolios is constrained by our requirement that

P(Threatagentperformsmassdisconnects>50MW)≤0.005.

Under this probabilistic constraint, we run the explicit enumeration algorithm to find sets of nondominated portfolios $Z_{ND}\left(B\right)$ for different budget levels B. This allows us to identify a sufficient level of investment yielding satisfactory improvements in the cybersecurity of our exemplary AMI system. Let $EV_{k}^{*}\left(B\right)$ be the lowest expected impact in the k‐th impact dimension that could be achieved by a Pareto‐optimal portfolio affordable under budget B, that is, $EV_{k}^{*}\left(B\right)\leq E\left(V_{k}\right)\left(z\right)$ for all $z\in Z_{ND}\left(B\right)$ with there being at least one $z_{k}^{*}\left(B\right)\in Z_{ND}\left(B\right)$ in existence, such that $EV_{k}^{*}\left(B\right)=E\left(V_{k}\right)\left(z_{k}^{*}\left(B\right)\right)$. Notice that there may be no feasible portfolio in $Z_{ND}\left(B\right)$ that can achieve the minimal expected impacts given budget B for all types of impacts simultaneously.

**FIGURE 6:**
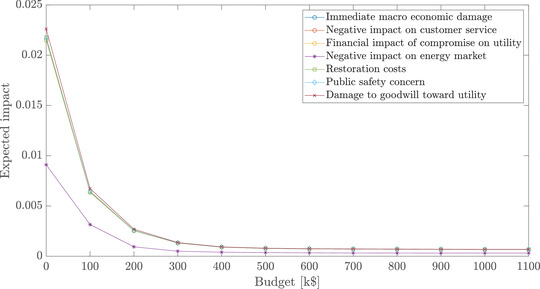
Expected impact of each impact criterion for different budget levels

**FIGURE 7:**
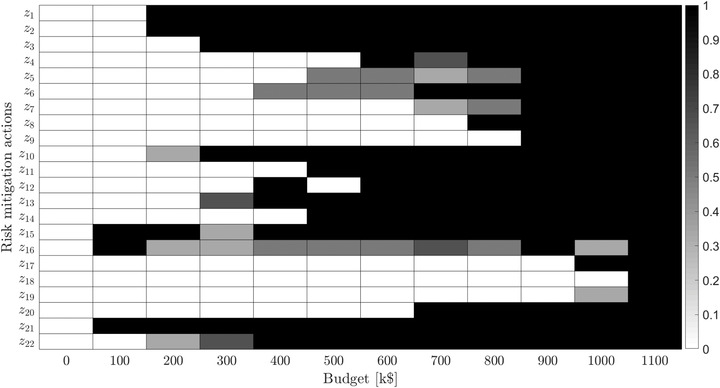
Core index map of mitigation actions for different budget levels

Fig. 6 displays $EV_{k}^{*}\left(B\right)$ as functions of B for impact dimensions k that are relevant to our example. It indicates that increasing the budget leads to more effective portfolios of mitigation measures and that, for each impact category, a minimal attainable expected impact can be achieved at 400 k$. However, a closer inspection of the values of the core indices of the considered measures plotted in Fig. 7 reveals that, for budgets close to 400 k$, tradeoffs in prioritizing impacts need to be made. For instance, all Pareto‐optimal portfolios affordable for a budget of 500 k$ contain measures $a_{1}-a_{3}$, $a_{10}$, $a_{11}$, $a_{13}-a_{15}$, $a_{21}$, and $a_{22}$, but each of the measures $a_{5}$, $a_{6}$, and $a_{16}$ belongs only to about 50% of portfolios. It is then up to the operators of our hypothetical system to decide between two options: (1) to limit the number of individuals with privileged access to the network (measure $a_{16}$), which decreases the probability of *Mass disconnects*; or (2) to improve the firewall (measures $a_{5}$ and $a_{6}$), making it more difficult for the threat agent to obtain *Credentials for meter disconnect function*, and thus reducing the likelihood not only of *Mass disconnects* but also of *Drastic rise in electricity usage* and *Self‐test failure messages*. Finally, increasing the budget to 900 k$ eliminates the need for these tradeoffs, as all three measures can now be afforded.

## 5 DISCUSSION

In this article, we proposed a method for developing a BN model for cyber risk assessment and management using elements of qualitative assessment frameworks that are based on expert judgment and scoring systems. It is important to point out that although our quantitative approach improves on certain of the limitations of qualitative methods, it nevertheless inherits, to a certain degree, the weaknesses of the underlying scoring systems.

The case problem of improving the cybersecurity of the AMI infrastructure, used as an illustration throughout this article, is based on the NESCOR studies (Electric Power Research Institute, 2015a, 2015b), which envisioned the use of partial impact scores (cf. Table S1), expressed on the common dimensionless scale with values of 0, 1, 3, or 9. The purpose of this scale was to facilitate the aggregation of partial impact scores into an overall impact score. When the scale is used to define states of the value nodes in the BN, however, it may have a strong influence on the shape of the Pareto front. The choice of different impact scales may lead to different conclusions (Hämäläinen & Lahtinen, 2016). To improve the realism of the analysis, we advise the quantification of impacts using their natural scales, for example, to express actual costs in monetary terms for the criterion *Restoration costs*.

Similarly, the results of our analysis are influenced by the assignment of occurrence probabilities to the events constituting attack scenarios, which could be a troublesome task. Analysis of data on successful and unsuccessful attacks could provide estimates of occurrence probabilities for events of a repetitive nature. Historical data, however, provide no information on the probabilities of potential but as yet unobserved exploits of cybersecurity vulnerabilities (Paté‐Cornell et al., 2017). Nevertheless, based on expert knowledge or predictive modeling, subjective and imprecise assignments can be made of the occurrence probabilities of rare or unique events, and these can be meaningfully combined with frequency‐based probability estimates within a Bayesian framework (Flage et al., 2016). Moreover, this framework allows estimates of occurrence probabilities to be updated as and when evidence of new attempted exploits become available (Jensen, 2001). The BN may also facilitate the modeling of unknown cybersecurity risks, and may make it possible to model a zero‐day threat as a type of attack action that may trigger any asset compromise. Furthermore, BN‐based models of cybersecurity can be extended to represent both human‐induced and natural hazards (e.g., severe weather conditions), which may aggravate the vulnerabilities of a cyber‐physical system, for example, a power grid (Ciapessoni et al., 2016).

In this article, we addressed the problem of finding optimal portfolios of “static” measures that reduce the expected impacts of cyberattacks. Yet, empirical studies (Holm, 2014) and BN‐based cybersecurity models (Zhang et al., 2015) both indicate that the expected time to system compromise decreases with the number of intrusions, eventually rendering any standing cybersecurity arrangement obsolete. This is because threat agents eventually gain experience at exploiting vulnerabilities they are aware of and discover new ones. Frigault et al. (2008) recognize that the security of a system depends not only on its current state, but also on the history of past intrusions. They also demonstrate how dynamic BNs can model the evolving cybersecurity condition of a system.

Dynamic management of cybersecurity conditions poses further challenges to the system operators in addition to setting up a “static” portfolio of security measures. System operators need to detect intrusions, accurately monitor the state of system security in real time, and optimally respond to the developing situation in case of an attack. The Bayesian framework discussed in this article can be used to address these challenges. Modelo‐Howard et al. (2008) propose a BN‐based method for optimally deploying intrusion detectors, while Peng Xie et al. (2010) discuss the use of BNs to infer in real time the actual state of system cybersecurity based on evidence and monitoring. Optimal response to an ongoing attack was discussed by Poolsappasit et al. (2012), who used a BAG with binary uncertainty nodes to model consecutive stages of attacks. They also proposed a genetic algorithm to approximate the set of Pareto‐optimal actions that counter the attack efficiently. Our BN model (Section 3) offers a more fine‐grained representation of system states, as it allows for uncertainty nodes having more than two states. Additionally, the implicit enumeration algorithm proposed in Section 4 allows the set of Pareto‐optimal response strategies to be computed (not just approximated). Moreover, this algorithm can readily be adapted to the dynamic BN setting. Mancuso et al. (2019) demonstrated its usefulness for optimal dynamic handling of contingencies in industrial processes.

Indeed, our next step will be to develop a dynamic BN‐based counterpart to the “static” model discussed in this article. Dynamic BN models of system cybersecurity with an explicit temporal dimension can be further extended. One direction is to represent the defender's beliefs about the attacker's actions and intentions to allow for an adversarial risk analysis (Banks et al., 2015; Insua et al., 2021). Another important direction is the modeling of cyber resilience (Gisladottir et al., 2016), understood as the system's ability to deliver its intended outcome despite adverse cybersecurity incidents.

We conclude this section with some remarks on how our framework could be operationalized in real‐world applications. As with other formal and quantitative risk analysis and management frameworks, taking advantage of the full potential of our approach would require considerable gestation time and efforts to adequately tailor it to the actual system under consideration. For example, an organization in charge of protecting a large‐scale cyber‐physical system, such as an electric power grid, may require a dedicated project of several person‐months, involving consultations with cybersecurity experts and systems operators to implement our framework. Typically, the process of developing a quantitative model starts with a high‐level risk assessment that focuses on a series of 1,0‐20 cybersecurity risks and a similar number of potential cybersecurity measures for a few critical subsystems responsible for specific functions (e.g., AMI system in case of power grids). Then, a more in‐depth analysis of identified risks based on attack trees would follow. To better understand the mechanisms of these threats, it is often helpful to use attack trees with three types of nodes: (1) attack actions (e.g., launch of Denial of Service attack); (2) the consequences of the attacks on the ICT assets (e.g., server unavailable, data leaked); and (3) the consequences of (1) and (2) for physical, human, or business assets (e.g., disconnection, safety event, monetary loss). Completion of such a high‐level assessment is a good starting point for using our framework. Once the high‐level implementation of our model is consolidated, it could then be tested, improved, and expanded in the subsequent iterations of the analysis. The modularity of BN models comes in handy for this process, as updates after adding new nodes or changes to individual CPTs are straightforward (thanks to the chain rule).

## 6 CONCLUSIONS

In this article, we addressed the problem of designing a comprehensive and quantitative model to (1) assess the cybersecurity risk of cyber‐physical systems such as smart electric power grids, and (2) optimize the selection of security measures that minimize the expected impacts. The cornerstone of our approach to this problem is a holistic representation of the cybersecurity landscape of a system as a BN derived from attack trees. This model provides an intuitive probabilistic representation of dependencies between stage events of cyberattacks corresponding to exploits of specific vulnerabilities of the system. It allows for the calculation of probabilities of successful cyberattacks, represented by cascading events, as well as the evaluation of their expected impacts according to a set of distinct criteria. We use this Bayesian model to compute expected reductions in those impacts that are achieved by deploying different security portfolios, identified as ones that are Pareto‐optimal. We aimed to overcome three existing gaps that may result in suboptimal cybersecurity resource allocation. These gaps are salient in the most prevalent risk analysis methods used in management of security of cyber‐physical systems. One gap is the poor suitability of frameworks that are based on scores and risk matrices to deal with the increasing complexity of cyber threats. Another drawback is that the occurrence of multiple, potentially synergistic attacks is not modeled in most of the popular approaches. A third caveat is the narrow perspective of commonly used cost–benefit analysis. The usefulness of attack trees and BNs in modeling the cybersecurity of various kinds of systems has been widely acknowledged in the literature. In this article, we demonstrated how this well‐established tool of quantitative modeling could be built to represent an integrated picture of cyber threats to cyber‐physical systems, based on the attack trees of individual cyberattack scenarios, like those developed by NESCOR for electric systems. Next, we framed the task of finding optimal portfolios of security measures as a problem of simultaneous minimization of multiple expected impacts under budget, technical, and probabilistic constraints. We also proposed an explicit enumeration algorithm as an efficient way of solving this multiobjective optimization problem and of computing the set of Pareto‐optimal portfolios of security measures. Finally, we discussed the usefulness of the core index as a guideline for selecting a robust portfolio from a possibly large set of Pareto‐optimal ones. We concluded this article with a discussion of the applicability of our method. We demonstrated its usefulness for modeling the cybersecurity of electric power grids. However, we noted that the whole framework or its elements can be readily adapted to security problems of other cyber‐physical and ICT systems, or to the reliability problems of industrial systems in general.

## Supporting information

Table A1: NESCOR impact criteria with scoring system (EPRI, 2015b)
Table A2: NESCOR likelihood criteria with scoring system (EPRI, 2015b).
Table A3: Mitigation actions for scenario Authorised employee brings malware into system or network (EPRI, 2015a).
Table A4: Mitigation actions for scenario Threat agent exploits firewall gap (EPRI, 2015a).
Table A5: Mitigation actions for scenario Threat agent uses social engineering (EPRI, 2015a).
Table A6: Mitigation actions for scenario Threat agent obtains credentials for system or function (EPRI,2015a)
Table A7: Mitigation actions for scenario Threat agent gains access to network (EPRI, 2015a).
Table A8: Mitigation actions for scenario Reverse engineering of AMI equipment allows unauthorised mass control (EPRI, 2015a).

## Footnotes

1 For our scenarios, the set of oval nodes is a union of nodes of AMI.9 and AMI.27 attack trees and their common subtrees. They include both conditions and impacts, though with shortened names. Note that the event *Threat agent obtains credentials for the meter disconnect function* is shared between both AMI.9 and AMI.27 scenarios and is thus represented by a single node named *Credentials for meter disconnect function*. In addition, the events *Threat agent has headend credentials and initiates disconnect(s) at headend* and *Threat agent has business system credentials and initiates disconnect(s) at business system* in Fig. 1(a) are not considered in the integrated attack graph because it is sufficient for the threat agent to gain access to the network hosting the meter disconnect function and obtain the credentials necessary to cause possible voltage/frequency fluctuations with disconnected customers.

2 The algorithm was implemented in the C++ programming language and linked to *GeNIe Modeler*, a development environment for reasoning in graphical probabilistic models, developed by BayesFusion LCC and available at http://www.bayesfusion.com/.
