# Supplementary material for: A Bayesian Framework for the Analysis and Optimal Mitigation of Cyber Threats to Cyber‐Physical Systems
Source: Risk Anal. 2022 Mar 1;42(10):2275–90. doi: 10.1111/risa.13900 (PMC9790388; doi:10.1111/risa.13900)
Supplement: Supplementary file 1 — Table A1: NESCOR impact criteria with scoring system (EPRI, 2015b) Table A2: NESCOR likelihood criteria with scoring system (EPRI, 2015b). Table A3: Mitigation actions for scenario Authorised employee brings malware into system or network (EPRI, 2015a). Table A4: Mitigation actions for scenario Threat agent exploits firewall gap (EPRI, 2015a). Table A5: Mitigation actions for scenario Threat agent uses social engineering (EPRI, 2015a). Table A6: Mitigation actions for scenario Threat agent obtains credentials for system or function (EPRI,2015a) Table A7: Mitigation actions for scenario Threat agent gains access to network (EPRI, 2015a). Table A8: Mitigation actions for scenario Reverse engineering of AMI equipment allows unauthorised mass control (EPRI, 2015a). [file RISA-42-2275-s001.pdf]

SUPPLEMENTARY TABLES

Table A1: NESCOR impact criteria with scoring system (EPRI, 2015b).

Impact criterion	Scoring system
System scale	0: single utility consumer; 1: neighbourhood; 3: town or city; 9: potentially full utility service area and beyond.
Public safety	0: none; 1: 10-20 injuries possible; 3: 100 injured possible; 9: one death possible.
Workforce safety	0: none; 3: any possible injury; 9: any possible death.
Ecological concern	0: none; 1: local ecological damage such as localised fire or spill, repairable; 3: permanent local ecological damage; 9: widespread temporary or permanent damage to one or more ecosystems.
Financial impact of compromise	0: petty cash or less; 1: up to 2% of utility revenue; 3: up to 5 %; 9: greater than 5 %.
Restoration costs	0: petty cash or less; 1: up to 1% of utility organisation O&M budget; 3: up to 10%; 9: greater than 10%.
Generation capacity	0: no effect; 1: small generation facility off-line or degraded operation of large facility; 3: more than 10% loss of generation capacity for 8 hours or less; 9: more than 10% loss of generation capacity for more than 8 hours.
Energy market	0: no effect; 1: localised price manipulation, lost transactions, loss of market participation; 3: price manipulation, lost transactions, loss of market participation impacting a large metro area; 9: market or key aspects of market non operational.
Bulk transmission system	0: no effect; 1: loss of transmission capability to meet peak demand or isolate problem areas; 3: major transmission system interruption; 9: complete operational failure or shut down of the transmission system.
Customer service	0: no effect; 1: up to 4 hour delay in customer ability to contact utility and gain resolution, lasting one day; 3: up to 4 hour delay in customer ability to contact utility and gain resolution, lasting a week; 9: complete operational failure or shut-down of the transmission system.
Billing functions	0: none; 1: isolated recoverable errors in customer bills; 3: widespread but correctable errors in bills; 9: widespread loss of accurate power usage data.
Goodwill toward utility	0: no effect; 1: negative publicity but this does not cause financial loss to utility; 3: negative publicity causing up to 20% less interest in programs; 9: negative publicity causing more than 20% less interest in programs.
Immediate macroeconomic damage	0: none; 1: local businesses down for a week; 3: regional infrastructure damage; 9: widespread runs on banks.
Long term economic damage	0: none; 3: several years of local recession; 9: several years of national recession.
Loss of privacy	0: none; 1: 1000 or less individuals; 3: thousands of individuals; 9: millions of individuals.

Table A2: NESCOR likelihood criteria with scoring system (EPRI, 2015b).

Likelihood criterion	Scoring system
Skill required	0: Deep domain/insider knowledge and ability to build custom attack tools; 1: Domain knowledge and cyberattack techniques; 3: Special insider knowledge needed; 9: Basic domain understanding and computer skills.
Accessibility (physical)	0: Inaccessible; 1: Guarded, monitored; 3: Fence, standard locks; 9: Publicly accessible.
Accessibility (logical, assume have physical access)	0: High expertise to gain access; 1: Not readily accessible; 3: Publicly accessible but not common knowledge; 9: Common knowledge or none needed.
Attack vector (assume have physical and logical access)	0: Theoretical; 1: Similar attack has been described; 3: Similar attack has occurred; 9: Straightforward, for example script or tools available.
Common vulnerability among others	0: Isolated occurrence; 1: More than one utility; 3: Half or more of power infrastructure; 9: Nearly all utilities.

Table A3: Mitigation actions for scenario *Authorised employee brings malware into system or network* (EPRI, 2015a). Note: costs of mitigation actions are not realistic and are used for illustrative purposes only.

Index	Mitigation actions	Cost [k$]	Affected event(s)
1	Train personnel on possible paths for infection	30	Compromised mobile device
			Compromised computer peripherals
			Unintentional installation of malware
2	Maintain patches and antivirus	70	Compromised mobile device
			Compromised computer peripherals
			Unintentional installation of malware
			Intentional installation of malware
3	Test for malware before connection	50	Compromised mobile device
			Compromised computer peripherals

Table A4: Mitigation actions for scenario *Threat agent exploits firewall gap* (EPRI, 2015a). Note: costs of mitigation actions are not realistic and are used for illustrative purposes only.

Index	Mitigation actions	Cost [k$]	Affected event(s)
4	Implement configuration management	40	Intentional set of firewall rule that permits access between two networks
			Accidental set of firewall rule that permits access between two networks
5	Verify all firewall changes	60	Intentional set of firewall rule that permits access between two networks
			Accidental set of firewall rule that permits access between two networks
6	Require intrusion detection	30	Intentional set of firewall rule that permits access between two networks
			Accidental set of firewall rule that permits access between two networks
7	Require authentication to access firewall	50	Intentional set of firewall rule that permits access between two networks
			Accidental set of firewall rule that permits access between two networks

Table A5: Mitigation actions for scenario *Threat agent uses social engineering* (EPRI, 2015a). Note: costs of mitigation actions are not realistic and are used for illustrative purposes only.

Index	Mitigation actions	Cost [k$]	Affected event(s)
8	Conduct penetration testing periodically	70	Info from Internet
			Info from dumpster diving
			Info from other means
			Threat agent posing as trustworthy party
9	Train personnel on social engineering attacks	40	Threat agent posing as trustworthy party

Table A6: Mitigation actions for scenario *Threat agent obtains credentials for system or function* (EPRI, 2015a). Note: costs of mitigation actions are not realistic and are used for illustrative purposes only.

Index	Mitigation actions	Cost [k$]	Affected event(s)
10	Strong passwords	30	Crack of passwords
11	Encrypt communication paths	80	Capture of passwords on network or through keystroke logger
12	Protect against replay	60	Capture of passwords on network or through keystroke logger
13	Strong security questions	30	Reset passwords
14	Require multi-factor authentication	50	No assistance from authorised user
15	Use a token with PIN	20	Theft of an authentication token

Table A7: Mitigation actions for scenario *Threat agent gains access to network* (EPRI, 2015a). Note: costs of mitigation actions are not realistic and are used for illustrative purposes only..

Index	Mitigation actions	Cost [k$]	Affected event(s)
16	Limit individuals with privilege	30	Having privilege to access network hosting disconnect function
			Privilege to access a network connected to network hosting disconnect function
17	Isolate network	90	Privilege to access a network connected to network hosting disconnect function
18	Enforce restrictive firewall rules to access connected network	70	Path to gain privilege to access network hosting disconnect function
19	Require authentication to access connected network	40	Path to gain privilege to access network hosting disconnect function

Table A8: Mitigation actions for scenario *Reverse engineering of AMI equipment allows unauthorised mass control* (EPRI, 2015a). Note: costs of mitigation actions are not realistic and are used for illustrative purposes only..

Index	Mitigation actions	Cost [k$]	Affected event(s)
20	Remove unsecured development features	80	Reverse engineering of AMI meters
21	Include credentials in equipment design	50	Control of many devices simultaneously
22	Configure for least functionality	30	Control of many devices simultaneously

REFERENCES

Electric Power Research Institute. (2015a). *Analysis of selected electric sector high risk failure scenarios – version 2.0.* Retrieved from https://www.nevermoresecurity.com/2015/12/25/analysis-of-selected-electric-sector-high-risk-failure-scenarios-version-2-0/

Electric Power Research Institute. (2015b). *Electric sector failure scenarios and impact analyses – version 3.0.* Retrieved from https://www.nevermoresecurity.com/2015/12/25/electric-sector-failure-scenarios-and-impact-analyses-version-3-0/
